# *GraphAlignment*: Bayesian pairwise alignment of biological networks

**DOI:** 10.1186/1752-0509-6-144

**Published:** 2012-11-21

**Authors:** Michal Kolář, Jörn Meier, Ville Mustonen, Michael Lässig, Johannes Berg

**Affiliations:** 1 Institut für Theoretische Physik, Universität zu Köln, Zülpicher Straße 77, D-50937 Köln, Germany; 2 Institute of Molecular Genetics, Academy of Sciences of the Czech Republic, Vídeňská 1083, CZ-14220 Praha, Czech Republic; 3 Present address: Wellcome Trust Sanger Institute, Wellcome Trust Genome Campus, Hinxton, CB10 1SA, UK

**Keywords:** Graph alignment, Biological networks, Parameter estimation, Bioconductor

## Abstract

**Background:**

With increased experimental availability and accuracy of bio-molecular networks, tools for their comparative and evolutionary analysis are needed. A key component for such studies is the alignment of networks.

**Results:**

We introduce the Bioconductor package *GraphAlignment* for pairwise alignment of bio-molecular networks. The alignment incorporates information both from network vertices and network edges and is based on an explicit evolutionary model, allowing inference of all scoring parameters directly from empirical data. We compare the performance of our algorithm to an alternative algorithm, *Græmlin 2.0*.

On simulated data, *GraphAlignment* outperforms *Græmlin 2.0* in several benchmarks except for computational complexity. When there is little or no noise in the data, *GraphAlignment* is slower than *Græmlin 2.0*. It is faster than *Græmlin 2.0* when processing noisy data containing spurious vertex associations. Its typical case complexity grows approximately as $O(N^{2.6})$.

On empirical bacterial protein-protein interaction networks (PIN) and gene co-expression networks, *GraphAlignment* outperforms *Græmlin 2.0* with respect to coverage and specificity, albeit by a small margin. On large eukaryotic PIN, *Græmlin 2.0* outperforms *GraphAlignment*.

**Conclusions:**

The *GraphAlignment* algorithm is robust to spurious vertex associations, correctly resolves paralogs, and shows very good performance in identification of homologous vertices defined by high vertex and/or interaction similarity. The simplicity and generality of *GraphAlignment* edge scoring makes the algorithm an appropriate choice for global alignment of networks.

## Background

The advent of high-throughput techniques has generated new types of large-scale molecular interaction data, conveniently represented by graphs or networks. Examples include metabolic networks formed by enzymes and metabolites [1], gene co-expression networks with edges between pairs of genes indicating a certain correlation between their expression levels [2], residue contact maps as representations of protein structures [3,4], and protein-protein interaction networks, where edges between vertices indicate a physical interaction between proteins [5]. For an introduction, see reference [6].

Cross-species analysis of bio-molecular networks aims to identify sub-networks which are evolutionarily conserved as well as network parts that have evolved rapidly. Similarly to comparison of biological sequences [7], alignment of biological networks is an important tool for quantitative evolutionary studies [2,8-16]. However, such alignment poses a challenging computational problem, which goes beyond the well-established concepts and methods of sequence alignment and of subgraph matching (isomorphism) [17]. It involves an evolutionary process in which a pair of networks derives from a common ancestor (which accounts for a certain degree of similarity), and each network has since evolved independently (which results in edge changes, vertex changes, and vertices losing their alignment partner).

**Figure 1:**
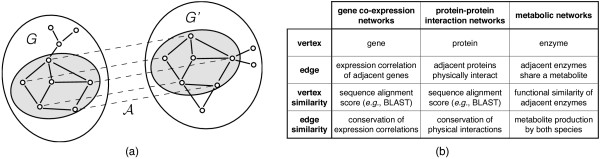
**Graph alignment.a)** An alignment A between two graphs is an injective one-to-one mapping (indicated by dashed lines) between the vertices of two graphs (see text). **b)** The interpretation of vertices and edges depends on the type of biological networks in comparison.

Here, we define the alignment of two graphs as an injective one-to-one mapping from a subset of vertices of one graph to vertices of the other graph, see Figure 1a. An alignment of vertices also induces the alignment of edges; the edge in one network is said to be aligned to the edge in the other network if the vertices they connect are aligned to one another. The aim of a *graph alignment* is to align vertices that descend from a common ancestor.

Several graph alignment methods have been proposed towards this goal, based on three main ideas: The alignment can be based on the similarity of vertices, and map vertices onto each other that, e.g., share a certain sequence similarity (if vertices represent genes or proteins) or if aligned enzymes catalyze the same reaction (if vertices represent enzymes in a metabolic network). This approach allows identification of ancestral networks [14], network parts enriched in conserved edges [10,12,16], or selection between paralogous genes [13].

A second and complementary approach focuses on the topology of the graphs and disregards sequence information or other properties of the vertices. It searches for similar topological structures in two graphs, for instance by maximizing the number of aligned edges. This approach has been used, for example, to detect common regulatory motives in gene regulatory networks [18,19] or to perform global network alignment [20].

A third strategy relies *both* on information encoded in vertices and in edges. This “hybrid” and more comprehensive approach compares graphs based on the evolution of both vertices and edges. The key problem is the relative weight given to the similarity of vertices and to the similarity of edges when constructing the alignment. Several algorithms have been proposed [11,21-27], which generally use ad hoc scoring parameters. Two exceptions are *GraphAlignment*[28] and *Græmlin 2.0* (hereafter *Græmlin*, [22]), which use parameters inferred from a training set or from an initial alignment of high-fidelity vertices (*Græmlin, GraphAlignment*), or in an iterative scheme (*GraphAlignment*). Here we describe a software package implementing the GraphAlignment algorithm.

The scoring parameters may indeed be inferred from a training dataset formed by a library of known orthologous genes and their interactions. This approach would be conceptually similar to the inference of the BLOSUM matrices [29] used for biological sequence comparison. As bio-molecular networks differ in many aspects, including experimental techniques and post-processing methods, no such parametrisation is available for their comparison. The parameters, however, can be also inferred from the actual data being aligned, similarly to the inference of the optimal affine gap penalties from the sequences being compared [30,31]. The ability to infer principled scoring parameters directly from the data is essential.

Further methods are developed that incorporate additional information resources to perform network alignment. The global network alignment method PINALOG [32] incorporates functional annotation of proteins in addition to their sequence and network topology. DOMAIN algorithm uses protein domains, rather than proteins, to form the interaction network [33]. Several above mentioned methods perform also multiple-species alignment and either use or infer phylogeny (e.g., [20,22,34]). Methods for querying large networks for small subgraphs, e.g, pathways or protein complexes, have been also developed [35-37], reviewed in [38].

*GraphAlignment* differs from the above approaches [11,21-27] by two key features: (a) An explicit model of network evolution is used to infer alignment parameters from the data. (b) Based on this evolutionary model, networks are aligned using a probabilistic scoring system. We compare our software and *Græmlin* as the only algorithms that can automatically score both sequence and network information. To that end we perform the simplest task, pairwise alignment.

For case studies applying our approach to mammalian gene co-expression networks and to herpesviral protein-protein-interaction networks, see [28] and [31]. An overview of related methods for probabilistic network analysis is given in ref. [39].

## Implementation

The input of the algorithm are two networks, and mutual similarities of their vertices. The algorithm treats the networks *G* and *G*^*′*^ symmetrically, thus comparison of *G* with *G*^*′*^ will result in the same alignment as comparison of *G*^*′*^ with *G*. Each network *G* is represented by an adjacency matrix **A**, whose entries *A*specify the edge between vertices i and j: The entries of the adjacency matrix may be binary, with *A*_*ij*_ = 1 indicating the presence of an edge between i and j, and *A*_*ij*_ = 0 its absence. They may be continuous, e.g., to describe weighted edges in gene co-expression networks. Adjacency matrices may be symmetric, thus describing undirected networks (e.g., gene co-expression networks), or asymmetric for directed networks (e.g., metabolic networks). The mutual similarity between vertices in the two networks is specified by matrix **Θ**, whose entries $\theta_{ii^{\prime }}$ quantify, for example, the overall sequence similarity between the gene represented by vertex *i* in one network and the gene represented by vertex i^′^ in the other. Any other measure of the vertex similarity is possible and may be given in arbitrary units (Figure 1b). The algorithm will infer appropriate scoring automatically based on available data.

The alignment scoring is based on an explicit model which incorporates evolutionary dynamics of both edges and vertices. We first focus on the evolutionary dynamics of the edges. Consider a pair of vertices *i,j* in one network and its orthologs *i*^′^,*j*^′^ in the second network. At speciation, the edge states a ≡ *A*_*ij*_ and $a^{\prime }\equiv A_{i^{\prime }j^{\prime }}^{\prime }$ in the two networks take on the same value. Subsequently, their correlation will decay and the joint probability Q_τ_(a,a^′^) will tend to a product of independent probabilities P(a)P^′^(a^′^) in the limit of large times τ. (See [28] for an explicit model based on the Fokker-Planck equation.) The corresponding log-likelihood score contribution from the pair of edges

(1) $$s_{\text{edge}}(a,a^{\prime })\equiv \log\left(\frac{Q_{\tau }(a,a^{\prime })}{P(a)P^{\prime }(a^{\prime })}\right)$$

tends to zero in the limit τ→∞, as then the edge states carry no information on their shared ancestry, and, hence, the edges states *a* and *a*^′^ carry no information on whether *i* should be aligned with *i*^′^and *j* with *j*^′^.

Analogous considerations for the evolutionary dynamics of the similarity of vertices leads to a scoring function for vertex similarity [28,31]: at speciation, vertex *i* in one network and its ortholog *i*^′^ in the second network do not differ. With increasing time τ since speciation, their vertex similarity *θ* will decrease and the distribution function $Q_{\tau }^{o}(\theta )$ will approach some background distribution P(*θ*). Likewise, with divergence of the two networks, the distribution function $Q_{\tau }^{u}(\theta )$ of the similarities $\theta_{ij^{\prime }}$ between unrelated vertices *i* and *j*^′^will approach P(*θ*). As τ→∞, the corresponding log-likelihood scores

(2) $$s_{\text{aligned}}(\theta_{ii^{\prime }})\equiv \log\left(\frac{\overset{o}{\underset{\tau }{Q}}(\theta_{ii^{\prime }})}{P(\theta_{ii^{\prime }})}\right),$$

which reflects vertex similarity of the orthologs *i* and *i*^′^, and

(3) $$s_{\text{not-aligned}}(\theta_{ij^{\prime }})\equiv \log\left(\frac{\overset{u}{\underset{\tau }{Q}}(\theta_{ij^{\prime }})}{P(\theta_{ij^{\prime }})}\right),$$

with j^′^ ≠ i^′^, which weighs the presence of vertex similar pairs that are not orthologous, tend to zero, and the vertex similarities $\theta_{ii^{\prime }}$ and $\theta_{ij^{\prime }}$ convey no information on alignment of *i* and *i*^′^. The background distribution P(*θ*) may be obtained as the distribution of vertex similarities between vertices that emerged or disappeared in one of the networks after the speciation. The similarity of vertices itself may be evaluated as sequence similarity for vertices representing genes or proteins (in gene co-expression networks and protein-protein interaction networks, respectively) or by the measure of functional similarity for vertices representing enzymes (in metabolic networks).

Given an alignment A , the total alignment score $S(A)=S_{e}(A)+S_{v}(A)$ is formed by contributions from all aligned vertices and edges. The edge score $S_{e}(A)$ sums contribution of aligned edges:

(4) $$S_{e}(A)=\underset{\left(i,j\right)}{\sum }s_{\text{edge}}\left(A_{\mathrm{ij}},A_{A(i)A(j)}^{\prime }\right).$$

The vertex score $S_{v}(A)$ sums contributions from the aligned vertices and the contributions from the pairs of vertices that are not aligned [28,31]:

(5) $$S_{v}(A)=\underset{i}{\sum }s_{\text{aligned}}(\theta_{iA(i)})+\underset{i,j^{\prime }\neq A(i)}{\sum }s_{\text{not-aligned}}(\theta_{ij^{\prime }}).$$

The parameters of the scoring function, i.e, s_edge_, s_aligned_ and s_not-aligned_, depend on the evolutionary dynamics of both edges and vertices since speciation. To infer these parameters from the data, we use a simple iterative approach [28]: Starting with an initial alignment, parameters are estimated so that the likelihood of the alignment is maximised. The algorithm then iterates the steps of (i) aligning the graphs using the estimated parameters and (ii) estimating the maximum likelihood parameters until convergence. Upon convergence, the algorithm returns both the optimal scoring parameters and the corresponding best alignment of the networks. The package GraphAlignment features built-in functions that establish the maximum-likelihood scoring parameters according to this scheme. The ability to find the appropriate scoring parameters from the studied graphs is unique to GraphAlignment, with a notable exception of Græmlin [22].

To find high-scoring graph alignments in step (i), we use an iterative heuristic described in [28]. This procedure is based on mapping to the quadratic assignment problem, solved iteratively by calls to a linear assignment solver, with added noise to help the alignment to escape from local score maxima, as in simulated annealing [40].

## Results and discussion

In Berg and Lässig [28] and Kolář et al. [31], our algorithm has been applied to gene co-expression networks and small protein-protein interaction networks. Here, we concentrate on evaluation of the computational complexity of the algorithm and comparison of its accuracy to the Græmlin algorithm [22], which is the only other algorithm able to infer principled scoring parameters automatically. We use both simulated and empirical bio-molecular data.

### Alignment of simulated networks

While experimental data provide the ultimate test set for the algorithms, and we will use them in the following section, we do not know the true evolutionary history of the networks and thus, we cannot assess the accuracy of the aligners fully. To that end we use simulated data. In the numerical experiment, pairs of orthologous vertices (orthologs) are assigned from the outset and, depending on the level of divergence, may have retained their vertex similarity (vertex homologs), interaction similarity (topological homologs or analogs) or both.

GraphAlignment and Græmlin are able to infer the scoring parameters either from a training set of known orthologous genes and their interactions or from some valid initial alignment of the actual network data being aligned. Here, we concentrate on the latter option. Both algorithms are given the same initial alignment of the networks that is formed by vertices with high vertex and topological similarity, and the parameters are inferred from this initial alignment.

We assess the computational cost and accuracy in three different scenarios which test three different aspects of the algorithms. In all the scenarios, we construct pairs of networks which contain 80% of orthologous vertices and 50% of all possible edges present. In scenario (i) we compare two networks with a substantial proportion of vertex homologs and a smaller set of analogous vertices, i.e., vertices that do not have any vertex similarity, yet they are, by their interactions, well anchored to the subnetworks consisting of vertex-orthologous vertices. Thus this scenario tests the ability of the algorithm to identify analogous vertices by properly evaluating the edge (interaction) similarity. We implement the scenario (i) by networks with 60%-interaction similarity between the orthologous pairs and with 62.5% of the orthologous pairs (50% of all vertices) having also a high vertex similarity. The interaction terms are randomly chosen from a uniform distribution and may be interpreted as edge weights or probabilities of the edge existence. We also assessed the scenario (i) with interaction terms selected from a normal distribution and obtain similar results (Additional file 1). An example of the corresponding Θ(i,i^′^) matrix of vertex similarities and correlation matrix of interaction similarities is given in Additional file 1: Figure S3(i, ia).

In scenario (ii), we test whether the algorithm is able to decide on an ortholog between two paralogous vertices. Specifically, we ask whether the algorithm is able to decide between two vertices in G^′^ with equal vertex similarity to i in G, one of which has also interaction similarity with i (the true ortholog) and the other shares no interactions (the spurious ortholog). We implement this scenario similarly to scenario (i) with 12.5% of the orthologs (10% of all vertices) having a paralog with no topological similarity. An example of the corresponding similarity structures is given in Additional file 1: Figure S3(ii).

**Figure 2:**
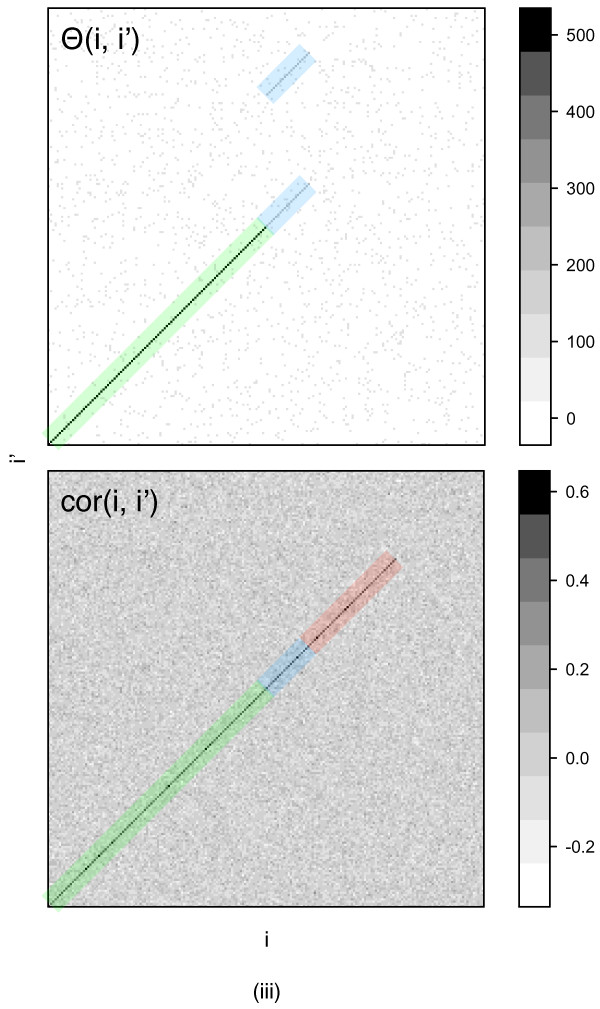
**Matrix of vertex similarities*Θ(i,i***^***′***^**) (top) and matrix of correlations between the edge weights of vertices*i*in*G*** and ***i***^***′***^***inG***^***′***^**(correlation of i’th column of*A*and*i***^***′***^**’th column of*A***^***′,***^***cor(i,i***^***′***^**), bottom) for the scenario (iii) and network size*N = 200.*** The optimal alignment of the two networks aligns the n-th vertex of *G* to the n-th vertex of *G*^*′*^. Half of the diagonal terms represents truly orthologous vertices with both vertex and topological similarity (highlighted in green). The other 10% of vertices *i* in *G* (highlighted in blue) have two possible vertex similar partners in network *G*^*′*^, one of them with a strong topological match (the true ortholog) and the other with no match (the spurious ortholog). Next, there are 20% of vertices with no vertex similarity but strong topological similarity (analogs, highlighted in red). Scattered off-diagonal terms in *θ* model spurious weak vertex similarities in the data.

Scenario (iii) derives from scenario (ii) but adds spurious weak vertex similarity between randomly chosen pairs of vertices. Thus, this scenario tests the robustness of the algorithms to intrinsic noise in the biological data. An example of the corresponding similarity structures is given in Figure 2.

#### Computational complexity

**Figure 3:**
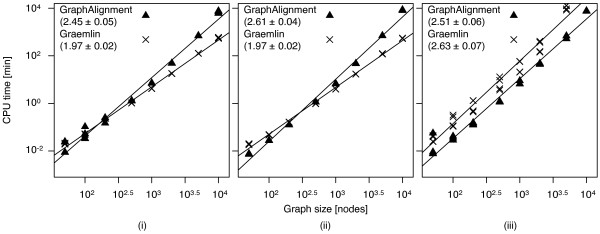
**Computational complexity of the *G*raphAlignment and *G ræmlin* algorithms.** The scaling parameters estimated from the best power law fit of the data are given in the panels for the scenarios (i-iii). While the computational cost of *G*raphAlignment remains constant in all the scenarios, *G*ræmlin’s performance deteriorates with addition of spurious weak vertex similarities in scenario (iii).

To evaluate the typical computational costs of *GraphAlignment* and *Græmlin*, we generate pairs of symmetric random networks of the same size, *N*∈[50,10^4^], and the corresponding similarity structures. Then, we test the two algorithms on the same dataset and measure the total CPU time used to fit the scoring parameters and to find the optimal graph alignment. Both algorithms are run on a Linux box with Intel Xeon at 3GHz with standard parameters (*GraphAlignment*: Scoring parameters are estimated by built-in functions from the initial alignment of the orthologs with high vertex similarity and the algorithm is run with standard settings. *Græmlin 2.0*: Scoring parameters are estimated according to the README file using the same set of vertices as in *GraphAlignment*. The algorithm is run with standard settings. For the code used, see Additional file 1: Figures S1 and S2). The results are summarised in Figure 3. In scenarios (i) and (ii) *Græmlin*’s computational costs scale roughly quadratically ( $O(N^{1.97\pm 0.02})$) with the network size *N*, while *GraphAlignment*’s costs grow as $O(N^{2.45\pm 0.05}))$ and $O(N^{2.61\pm 0.04})$, respectively. The algorithms finish the calculations of networks with the size *N* = 500 within the same time period, with *Græmlin* being faster on larger networks and *GraphAlignment* on smaller ones. However, addition of the spurious weak vertex similarities in scenario (iii) severely compromises *Græmlin*’s performance by changing its typical-case complexity to $O(N^{2.63\pm 0.07})$, so that a calculation for networks of size *N* = 10^4^ has not been concluded in two weeks. The performance of *GraphAlignment* remains good, with all calculations finished within a week of CPU time.

The typical-case computational cost of *GraphAlignment* is smaller than its theoretical worst-case complexity, which is dominated by the computational costs of the linear assignment solver [41] and by conversion of the edge score to an instance of the linear assignment problem. The overall worst-case complexity of the algorithm is $O(N^{3})$.

#### Accuracy

Both algorithms studied here rely on the initial alignment of high-fidelity vertices, which in our numerical experiment are represented by the orthologs with high vertex and topological similarity, and on inference of the scoring parameters from this initial alignment. Thus, it is not surprising that both algorithms correctly identified these orthologs in virtually all cases (corresponding to green diagonals in Figure 2). The algorithms differ, however, in their ability to align analogs (orthologs with no vertex similarity and high topological similarity in scenarios (i-iii)) and to decide on the true ortholog between two paralogs in scenarios (ii) and (iii).

While *GraphAlignment* performs pairwise alignment of the networks and its results are straightforwardly interpretable, *Græmlin* groups the vertices from both networks into equivalence classes which may contain several vertices from each network. When interpreting *Græmlin*’s results, there are two options to consider the vertices correctly aligned. We can consider the matching vertices of the two networks to be correctly aligned when they are in the same equivalence class *a*nd there is no other vertex in the class (*the strict rule*), or we can consider them correctly aligned whenever they are in the same equivalence class (*the relaxed rule*). It is worth noting that in scenarios (ii) and (iii) the relaxed rule will consider the vertex correctly aligned even if the equivalence class contains both its homologous paralogs and the alignment actually does not decide on the correct partner. A vertex is considered misaligned when it is in an equivalence class (of size greater than 1) where its matching vertex is not present. If the class contains vertices from a single graph only, these are not considered misaligned.

**Figure 4:**
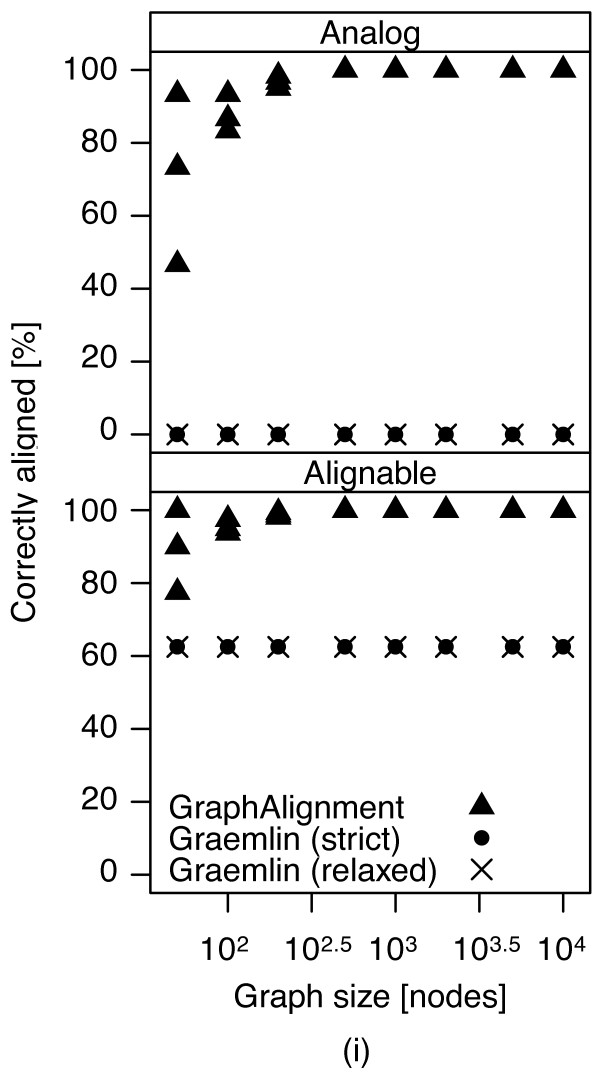
**Accuracy of*GraphAlignment*and*Græmlin*in scenario (i).** While *GraphAlignment* aligns a large proportion or all analogous vertices, *G*ræmlin aligns only the pairs of orthologous vertices with both vertex and topological similarity and no other vertices. The proportion of 62*.*5*%*corresponds to the fraction of those orthologs (50% of all vertices) among all orthologous vertices (80% of all vertices).

In scenario (i), there are only three types of vertex pairs: pairs with strong vertex and topological similarity, pairs with topological similarity only and pairs with no similarity between the networks. The first two groups, the orthologs, can be aligned thanks to the information stored in the similarity matrix **Θ** and the correlations of the adjacency matrices *A* and *A*^*′*^, see Additional file 1: Figure S3. Thus we call them *alignable* vertices. It is not possible to align the other vertices as there is no information available on those vertices. Figure 4 shows the accuracy of the algorithms in scenario (i): *Græmlin*, according to both strict and relaxed rules, aligns only orthologs with both vertex and topological similarity and no other vertices. *GraphAlignment* aligns a large proportion of the analogous vertices and in the case of networks of size greater than 500, all of them. None of the algorithms misaligns any vertices.

**Figure 5:**
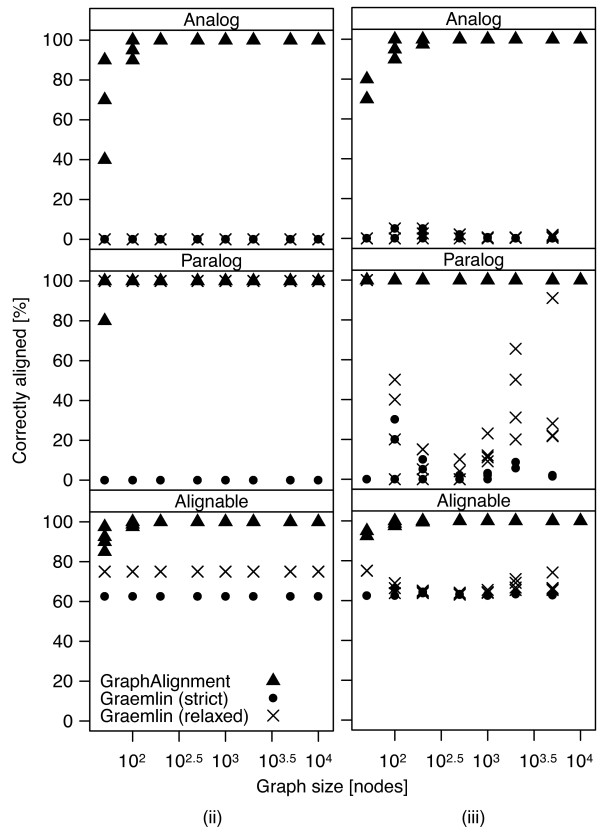
**Accuracy of *GraphAlignment *and *Græmlin *in scenarios (ii) and (iii).** While *GraphAlignment* correctly decides between paralogous genes, *Græmlin* creates equivalence classes that include both paralogs and their respective partner in the other network. The introduction of spurious weak vertex similarities does not influence *GraphAlignment* performance, yet it prevents *Græmlin* from forming the appropriate equivalence classes.

**Figure 6:**
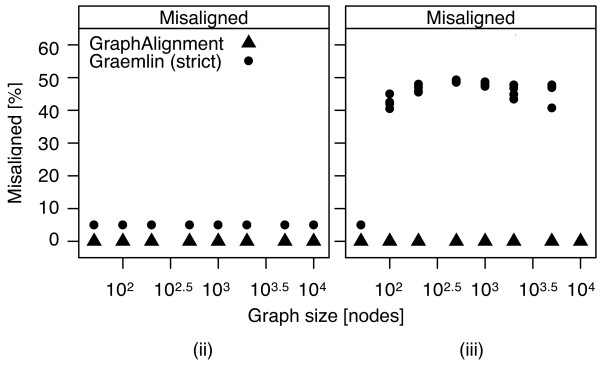
**Accuracy of *Græmlin *decreases upon introduction of spuriously similar vertex pairs in scenario (iii).***GraphAlignment* is not sensitive to the introduced noise. *Græmlin*, in addition to a decreased number of correctly aligned vertices (Figure 5), falsely aligns a substantial fraction of the vertices. The constant level of 5% misaligned vertices in (ii) corresponds to the paralogous vertices that are aligned in the correct equivalence class but are not the true matching vertices (the upper blue diagonal in Figure 2).

Paralogous vertices in scenario (ii) can be considered an easier task to resolve, as among *N* possible alignment partners, there are only two partners with some vertex similarity and, of them, just one also shares topological similarity with its ortholog. *GraphAlignment* aligns the matching vertices in virtually all tested instances of the problem. On the other hand, *Græmlin* correctly forms equivalence classes for the three vertex-similar vertices, as revealed by perfect performance according to the relaxed rule; however, it does not decide between the paralogous vertices as in the equivalence classes all three vertices are always present, Figure 5(ii). Also in the second scenario *GraphAlignment* does not misalign any vertex, Figure 6(ii), while *Græmlin* misaligns 5% of the vertices due to unresolved paralogous vertices.

Addition of the spurious terms into the vertex similarity matrix *θ* in scenario (iii) does not influence the accuracy of *GraphAlignment* but decreases accuracy of the *Græmlin* algorithm, which is not able to form the equivalence classes correctly anymore and misaligns many vertices, see Figures 5(iii) and 6(iii).

### Alignment of empirical bio-molecular networks

To compare the performance of *GraphAlignment* and *Græmlin* on diverse bio-molecular networks, we have downloaded publicly available datasets of bacterial and eukaryotic protein-protein interaction networks (PIN) and gene co-expression networks. We let the algorithms compare PIN of proteobacteria *Escherichia coli*, *Caulobacter crescentus* and *Campylobacter jejuni*, and of yeast *Saccharomyces cerevisiae*, mouse and human. Next, we employ the algorithms to compare gene co-expression networks of gamma-proteobacteria *Escherichia coli*, *Salmonella enterica* and *Shewanella oneidensis* and a firmicute, *Bacillus subtilis*. The specificity and coverage of the resultant alignments are tested against the orthologous groups defined in the eggNOG database v3.0 [42].

Protein sequences of all species have been downloaded from the eggNOG database. PIN of the bacterial species have been downloaded from the STRING database v9.0 [43]. Human and murine PIN have been obtained from the IntAct database v3.1 ( [44], accessed on August 6, 2012). Only high-confidence experimental interactions are kept (STRING: score ≥ 0*.*7, IntAct: miscore ≥ 0*.*35, no spoke-expanded interactions). To diversify the entering data, the PIN and protein sequences of human have been downloaded from the Additional file of the reference [45], and the yeast PIN and protein sequences from the Additional file of the reference [46] and the Saccharomyces genome database (http://www.yeastgenome.org,, accessed on August 8, 2012) [47], respectively.

To create the gene co-expression networks, we have downloaded large gene expression compendia of *Escherichia coli*, *Salmonella enterica* and *Bacillus subtilis* from the Colombos database ( [48], accessed on August 31, 2012). The database contains 2369, 925, and 397 carefully normalised expression profiles, respectively. Further, we use gene expression compendia of *Escherichia coli* and *Shewanella oneidensis* downloaded from the Many Microbe Microarrays Database (*M*^3*D*^, [49], accessed on September 6, 2012), which contain 907 and 245 expression profiles, respectively. Gene–gene co-expression levels are estimated by absolute Spearman rank correlation. Values lower than 0.5 are hard-thresholded to 0, except for the datasets from *M*^3*D*^, which are thresholded at 0.8 and 0.85, respectively. All final correlation coefficients are statistically significant (Storey’s *q* < 0*.*001). Only the genes detected in at least 75% of the profiles are evaluated.

The sequence similarity is estimated for each comparison by a pairwise local sequence alignment of protein sequences using BLAST [50]. All hits with e-value lower than 10^−10^ are considered. The BLAST scores are used as the measure of vertex similarity **Θ** provided to *GraphAlignment* and *Græmlin*. The orphan proteins/genes that both have no BLAST hit in the other species and are not connected in the bio-molecular network are not considered in the analysis. Table 1 summarizes the resultant networks.

**Table 1:** Bio-molecular networks used in the analyses

	**Protein-protein interaction networks**						
**Source**		**StringDB**		**IntAct**		**Ref. [**[46]**]**	**Ref. [**[45]**]**
**Species**	*ecoli*	*ccres*	*cjeju*	*mmusc*	*hsapi*	*scere*	*hsapi*
**Vertices**	822	477	369	7977	8984	2384	9141
**Edges**	1777	601	687	1594	26818	16070	41456
	**Gene co-expression networks**						
**Source**		**Colombos**		**M3D**			
**Species**	*ecoli*	*sente*	*bsubt*	*ecoli*	*sonei*		
**Vertices**	1219	1104	2212	2162	2358		
**Edges**	5589	4731	11181	4379	3823		

bsubt: Bacillus subtilis, ccres: Caulobacter crescentus, cjeju: Campylobacter jejuni, ecoli: Escherichia coli, hsapi: human, mmusc: mouse, scere: Saccharomyces cerevisiae, sente: Salmonella enterica, sonei: Shewanella oneidensis.

#### Computational complexity

**Figure 7:**
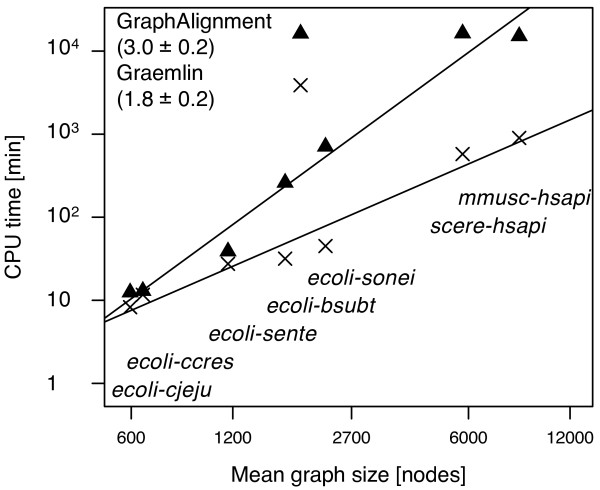
**Computational complexity of the *GraphAlignment *and *Græmlin *algorithms on empirical bio-molecular networks.** The scaling parameters estimated from the best power law fit of the data are given. Below the data points, the respective comparisons are indicated. For explanation of the abbreviations, see Table 1.

We evaluate the overall CPU time used by the algorithms to fit the scoring parameters and to perform the actual alignment. To define the training set for the parameter estimation, we find the eggNOG orthologous groups present in both aligned species. From these groups we randomly select one half. The proteins belonging to the selected orthologous groups and the interactions between them are then used as the training set. Both algorithms are allotted the same set and the scoring parameters are estimated by standard routines, as in case of the simulated networks. To align the networks, the algorithms run with standard settings, see Additional file 1: Figures S1 and S2. Figure 7 summarizes the computational complexity of the computations: As in the case of the simulated networks (scenarios (i) and (ii)), *Græmlin*’s computational costs scale roughly quadratically ( $O(N^{1.8\pm 0.2})$), while ***GraphAlignment***’s costs grow rather cubically as $O(N^{3.0\pm 0.2)})$. The algorithms finish the calculations on small bacterial networks within comparable intervals; *Græmlin* is significantly faster on larger eukaryotic networks.

#### Accuracy

To determine the quality of the resultant alignments, we estimate their sensitivity and coverage. As there is no gold standard with which to compare the results, we define *sensitivity* as the fraction of the aligned pairs, or *Græmlin* equivalence classes, which share the eggNOG orthologous group among all aligned pairs or classes. This measure of sensitivity is intrinsically biased, as the eggNOG orthologous groups are based on sequence comparison. Thus, the vertices which are orthologous, yet their sequences have diverged beyond recognition by the methods used to construct the eggNOG orthologous groups, do not contribute to this measure. We define *coverage* as the fraction of the eggNOG orthologous groups shared by the two species and correctly identified by the network alignment. Specifically, for *GraphAlignment*, let *NA* be the number of aligned pairs and *NC* be the number of the correctly aligned pairs in which the vertices (proteins or genes) belong to the same orthologous group as defined by eggNOG. Let *NO*be the total number of orthologous groups shared by the vertices of the networks being compared. Then, we define the sensitivity as *NC*/*NA* and coverage as *NC*/*NO*. For *Græmlin*, we define *NA*as the number of equivalence classes in which both species are represented. As in case of the simulated networks, we consider two rules for counting the number of correctly aligned equivalence classes *NC*: an equivalence class is correctly aligned either when all vertices are in the same eggNOG orthologous group *and* there is no vertex belonging to a different orthologous group in the class (*the strict rule*), or we consider the class correctly aligned whenever any two vertices belong to the same orthologous group (*the relaxed rule*). As the relaxed rule cannot decide between protein families, we will concentrate on the strict rule. Definition of the sensitivity and coverage remain the same.

We summarize the results on PIN in Table 2: On the bacterial networks *GraphAlignment* slightly outperforms *Græmlin* both in sensitivity and coverage, considering the strict rule. Both algorithms reach sensitivity of more than 65% and coverage of more than 90%. While comparing the eukaryotic PIN, *Græmlin* outperforms *GraphAlignment* on the IntAct-derived human and murine networks. Further, *GraphAlignment* significantly lags behind *Græmlin* comparing the human and yeast literature-based networks. Considering the contributions of the edge and node score, see Table 2, we see that the alignment provided by *GraphAlignment* is in that case dominantly driven by the edge score. This contrasts with the situation in comparing the other PIN networks, where the contributions are either even or dominated by the node score. The algorithm clearly overestimates the edge conservation rate between vertices with low sequence homology, which is inferred from the edge conservation rate between the orthologous vertices in the training set. That may have two reasons: Either the protein interaction data are biased in a way that is not compatible with the *GraphAlignment* Bayesian model, or different rates of interaction divergence occur between high-confidence orthologs (the training set) and proteins with low sequence similarity. Different rates of protein-protein interaction conservation depending on sequence similarity have indeed been documented recently [51]. The situation does not appear in the alignment produced by *Græmlin*, which places more weight on vertex similarity, as we saw in the previous section.

**Table 2:** **GraphAlignment and *Græmlin *performance on empirical bio-molecular networks**

**Comparison**	***Escherichia coli vs. Caulobacter crescentus***			***Escherichia coli vs. Campylobacter jejuni***		
**Algorithm**	**GraphAlignment**	**Græmlin**	**Blast BBH**	**GraphAlignment**	**Græmlin**	**Blast BBH**
**NA **	445	467	462	354	363	357
**NC**	319	309 (333)	333	247	241 (253)	253
**NO**	331	331	331	255	255	255
**NC / NA [%]**	71.7	66.2 (71.3)	72.1	69.8	66.3 (69.7)	70.9
**NC / NO [%]**	96.4	93.4 (101)	101	96.9	94.5 (99.2)	99.2
**Edge / vertex score**	2505 / 2774	-	-	2592 / 2253	-	-
**Comparison**	***Homo sapiens vs. Mus musculus***			***Homo sapiens vs. Saccharomyces cerevisiae***		
**Algorithm**	**GraphAlignment**	**Græmlin**	**Blast BBH**	**GraphAlignment**	**Græmlin**	**Blast BBH**
**NA**	7919	7907	7862	2369	1213	988
**NC**	5743	6327	6375	581	869 (882)	808
**NO**	6402	6402	6402	965	965	965
**NC / NA [%]**	72.5	80.0 (80.0)	81.1	24.5	71.6 (72.7)	81.8
**NC / NO [%]**	89.7	98.8 (98.8)	99.6	60.2	90.1 (91.4)	83.7
**Edge / vertex score**	2034 / 64661	-	-	20025 / 3963	-	-

Protein-protein interaction networks. For Græmlin, the values are calculated using the strict rule. Values obtained following the relaxed rule are given in parentheses. For GraphAlignment, the relative contributions of the edge and node score are also given. Results obtained using BLAST bidirectional best hit are provided for comparison.

When considering the gene co-expression networks, we observe very similar performance of *GraphAlignment* and *Græmlin*. The former algorithm provides better coverage (by at least 5%), while the latter shows slightly better sensitivity, with the exception of the comparison of *Escherichia coli* and *Salmonella enterica*, in which *GraphAlignment* has both better coverage and sensitivity. See Table 3 and Additional file 1: Table S1 for the summary of the results.

**Table 3:** **GraphAlignment and *Græmlin *performance on empirical bio-molecular networks**

**Comparison**	***Escherichia coli vs. Salmonella enterica***			***Escherichia coli vs. Bacillus subtilis***		
**Algorithm**	**GraphAlignment**	**Græmlin**	**Blast BBH**	**GraphAlignment**	**Græmlin**	**Blast BBH**
**NA**	624	687	662	585	459	401
**NC**	539	492 (562)	557	259	237 (296)	274
**NO**	543	543	543	284	284	284
**NC / NA [%]**	86.4	71.6 (81.8)	84.1	44.3	51.6 (64.5)	68.3
**NC / NO [%]**	99.3	90.6 (104)	103	91.2	83.5 (104)	96.5
**Edge / vertex score**	1453 / 4789	-	-	1979 / 2550	-	-

Gene co-expression networks. See Table 2 for details.

## Conclusions

Here we describe a software package for alignment of biomolecular networks based on a hybrid method developed in [28], *GraphAlignment*, and compare it to the algorithm *Græmlin 2.0*. We find advantages on both sides: the standalone *Græmlin* is able to perform multiple network comparisons and provides additional functionalities, e.g., clustering. As revealed on simulated data, ***GraphAlignment*** outperforms *Græmlin* in the use of interaction information for network alignment. We attribute the observed differences to the full use of interaction information: when an edge between a pair of aligned nodes is absent in both networks, *GraphAlignment* will typically reward the alignment of the nodes by a small score; *Græmlin* does not consider this piece of information. Consequently, *Græmlin* tends to align dense conserved clusters. This behaviour is advantageous for detection of such clusters, but may not be optimal in global alignment of sparse networks.

Comparison of empirical bacterial protein-protein interaction networks shows that *GraphAlignment* performs slightly better than *Græmlin* considering both sensitivity and coverage. Comparing the interaction networks of human and mouse based on the IntAct database, the situation is reversed. Moreover, we have observed limitations of the *GraphAlignment* algorithm in comparison of yeast and human protein-protein interaction networks, where the performance of the algorithm is decreased, most probably because the Bayesian scheme cannot deal with biased data or with the heterogenous rate of edge dynamics. On bacterial gene co-expression networks, *GraphAlignment* provides better coverage than *Græmlin*, while the sensitivity of both algorithms is similar. Considering the computational complexity, *GraphAlignment* is as efficient as *Græmlin* on small bacterial networks, while it lags significantly on large eukaryotic networks.

The simplicity and generality of *GraphAlignment* edge scoring makes this algorithm an appropriate choice for global alignment of networks. The underlying model is independent of the interpretation of edge weights, i.e., whether these weights represent probabilities of interaction between adjacent vertices or measure interaction strength. Since the algorithm is based on a well-defined evolutionary model, its parameters can be optimized by Bayesian methods. The *GraphAlignment* procedure of data input, estimation of scoring parameters and alignment of the networks is thoroughly documented in the package vignette, which also contains example sessions. Furthermore, we have shown that *GraphAlignment* is more robust to noise, an intrinsic factor of biological data, which is represented in our simulated data by spurious vertex similarities.

## Availability and requirements

The *GraphAlignment* algorithm is provided as an R package available from Bioconductor http://www.bioconductor.org and runs on all major platforms. Computationally intensive routines are coded in C. The software package can be used freely and with no restrictions for non-commercial purposes. It contains a code implementing the Jonker-Volgenant algorithm [41] to solve linear assignment problems. The code was written by Roy Jonker, MagicLogic Optimization Inc. and is copyrighted, 2003 MagicLogic Systems Inc., Canada. The code may be used freely for non-commercial purposes. For full details see the package vignette, the web page http://www.thp.uni-koeln.de/∼berg/GraphAlignment and the case studies [28,31].

## Competing interests

Authors declare no competing interests.

## Authors’ contributions

All authors contributed equally to the work. All authors read and approved the final manuscript.

## Supplementary Material

Additional file 1 **The Additional file 1 contains the codes used to generate the network instances and to find the optimal alignment by*GraphAlignment*and*Græmlin 2.0*, Figures S1 and S2.** Further, it contains Figure S3 with the matrix of vertex similarities *Θ*(*i*,*i*^*′*^)and the matrix of correlations between the edge weights of vertices *i* in *G* and *i*^*′*^ in *G*^*′*^ for the scenarios (i) and (ii). Figures S4 and S5 give the computational complexity and accuracy of the *GraphAlignment* and *Gæmlin* algorithms in scenario (ia) with the edge weights drawn from the normal distribution. Finally, Table S1 compares the *GraphAlignment* and *Græmlin* performance on empirical gene co-expression networks. Click here for file
