# Supplementary material for: GraphAlignment: Bayesian pairwise alignment of biological networks
Source: BMC Syst Biol. 2012 Nov 21;6:144. doi: 10.1186/1752-0509-6-144 (PMC3573967; doi:10.1186/1752-0509-6-144)
Supplement: Additional file 1 — The Additional file 1 contains the codes used to generate the network instances and to find the optimal alignment byGraphAlignmentandGræmlin 2.0, Figures S1 and S2. Further, it contains Figure S3 with the matrix of vertex similarities Θ(i,i′)and the matrix of correlations between the edge weights of vertices i in G and i′ in G′ for the scenarios (i) and (ii). Figures S4 and S5 give the computational complexity and accuracy of the GraphAlignment and Gæmlin algorithms in scenario (ia) with the edge weights drawn from the normal distribution. Finally, Table S1 compares the GraphAlignment and Græmlin performance on empirical gene co-expression networks. [file 1752-0509-6-144-S1.pdf]

Additional_file_1: GraphAlignment: Bayesian pairwise alignment of biological networks

Michal Kolář[1,2], Jörn Meier[1], Ville Mustonen[1,3], Michael Lässig[1]and Johannes Berg*[1]

[1]Institut für Theoretische Physik, Universität zu Köln, Zülpicher Straße 77, D-50937 Köln, Germany
[2]Institute of Molecular Genetics, Academy of Sciences of the Czech Republic, Vídeňská 1083, CZ-14220 Praha, Czech Republic
[3]Present address: Wellcome Trust Sanger Institute, Wellcome Trust Genome Campus, Hinxton, CB10 1SA, UK

Email: Michal Kolář - kolarmi@img.cas.cz; Jörn Meier - mail@ionflux.org; Ville Mustonen - vm5@sanger.ac.uk; Michael Lässig - lassig@thp.uni-koeln.de; Johannes Berg*- berg@thp.uni-koeln.de;

*Corresponding author

```
library(GraphAlignment);
sizes <- c(50, 100, 200, 500, 1000, 2000, 5000, 10000);

ex <- al <- vector("list", length = length(sizes));
names(ex) <- names(al) <- as.character(sizes);

## generate example instances (scheme (ia))
for (s in sizes) {
  size <- as.character(s);
  ex[[size]] <- GenerateExample(dimA = s, dimB = s, filling = 0.5, covariance = 0.6,
                symmetric = TRUE, numOrths = s / 2, correlated = seq(1, 0.8 * s));
  ex[[size]]$r <- 500 * ex[[size]];
}
save.image("generatedExamples.RData");

for (s in sizes) {
  size <- as.character(s);
  beta <- ceiling(max(abs(rnorm((1.7 * s)^2))));

  ## initial alignment
  pinitial   <- InitialAlignment(psize = 1.7 * s, r = ex[[size]]$r, mode = "reciprocal");

  ## scoring parameters
  linkParams <- ComputeLinkParameters(ex[[size]]$a, ex[[size]]$b, pinitial, lookupLink = seq(-2, 2, 0.5));
  nodeParams <- ComputeNodeParameters(dimA = s, dimB = s, ex[[size]]$r, pinitial,
                lookupNode = c(-100, 300, 600));

  ## optimal alignment
  al[[size]] <- AlignNetworks(A = ex[[size]]$a, B = ex[[size]]$b, R = ex[[size]]$r, P = pinitial,
                linkScore = linkParams$ls, selfLinkScore = linkParams$lsSelf, lookupLink = seq(-2, 2, 0.5),
                nodeScore1 = nodeParams$s1, nodeScore0 = nodeParams$s0, lookupNode = c(-100, 300, 600),
                bStart = beta, bEnd = 20 * beta, maxNumSteps = 20);
}
```

Figure S1: The code used to generate the network instances and to find the optimal alignment by *GraphAlignment*. Total execution time of fitting the score parameters and finding the alignment was measured by the R function system.time. The parameter *maxNumSteps* was set to 50 in comparison of actual bio-molecular networks and the look up tables were chosen to match the quartiles of actual data.

```
load("generatedExamples.RData");

for (s in c(50, 100, 200, 500, 1000, 2000, 5000, 10000)) {
  size <- as.character(s);

  ## name vertices of the two networks differently
  nA <- sprintf("1%06d", 1:s);
  nB <- sprintf("7%06d", 1:s);

  ## properties file
  sink("properties.txt");
  cat("blast_bitscore\tsynteny\tbest_bidirectional\n");
  rel <- which(diag(ex[[size]]$r > 0));
  for (r in rel)
    cat(nA[r], "\t", nB[r], "\t", ex[[size]]$r[r, r], "\t", 1, "\t", 1, "\n", sep = "");
  sink();

  ## traininig file
  sink("train.txt");
  for (r in rel)
    cat(nA[r], "\t", nB[r], "\n", sep = "");
  sink();

  ## networks files
  sink("network_a.net");
  cat("network_a\nfull\n");
  for (a1 in 1:s)
    for (a2 in 1:s) if (a1 >= a2)
      cat(nA[a1], "\t", nA[a2], "\t", ex[[size]]$a[a1, a2], "\n", sep = "");
  sink();

  sink("network_b.net");
  cat("network_b\nfull\n");
  for (b1 in 1:s)
    for (b2 in 1:s) if (b1 >= b2)
      cat(nB[b1], "\t", nB[b2], "\t", ex[[size]]$b[b1, b2], "\n", sep = "");
  sink();

  ## tree file
  sink("tree.txt");
  cat("(network_a:1,network_b:1)\n");
  sink();

  ## scoring parameters
  system(paste("../../graemlin -max-iterations 400 -alignment-training-set train.txt",
         "-alignment-params-out-file params.txt -treefile tree.txt -property-file properties.txt *.net"));

  ## optimal alignment
  system(paste("../../graemlin -no-cluster -alignment-params-file params.txt ",
         "-treefile tree.txt -property-file properties.txt *.net > alignment", size, ".txt", sep = ""));
}
```

Figure S2: The code used to read in the network instances and find the optimal alignment by *Græmlin*. Total execution time of fitting the score parameters and finding the alignment was measured by the R function system.time. *Græmlin 2.0* was compiled with the MaxPerf option.

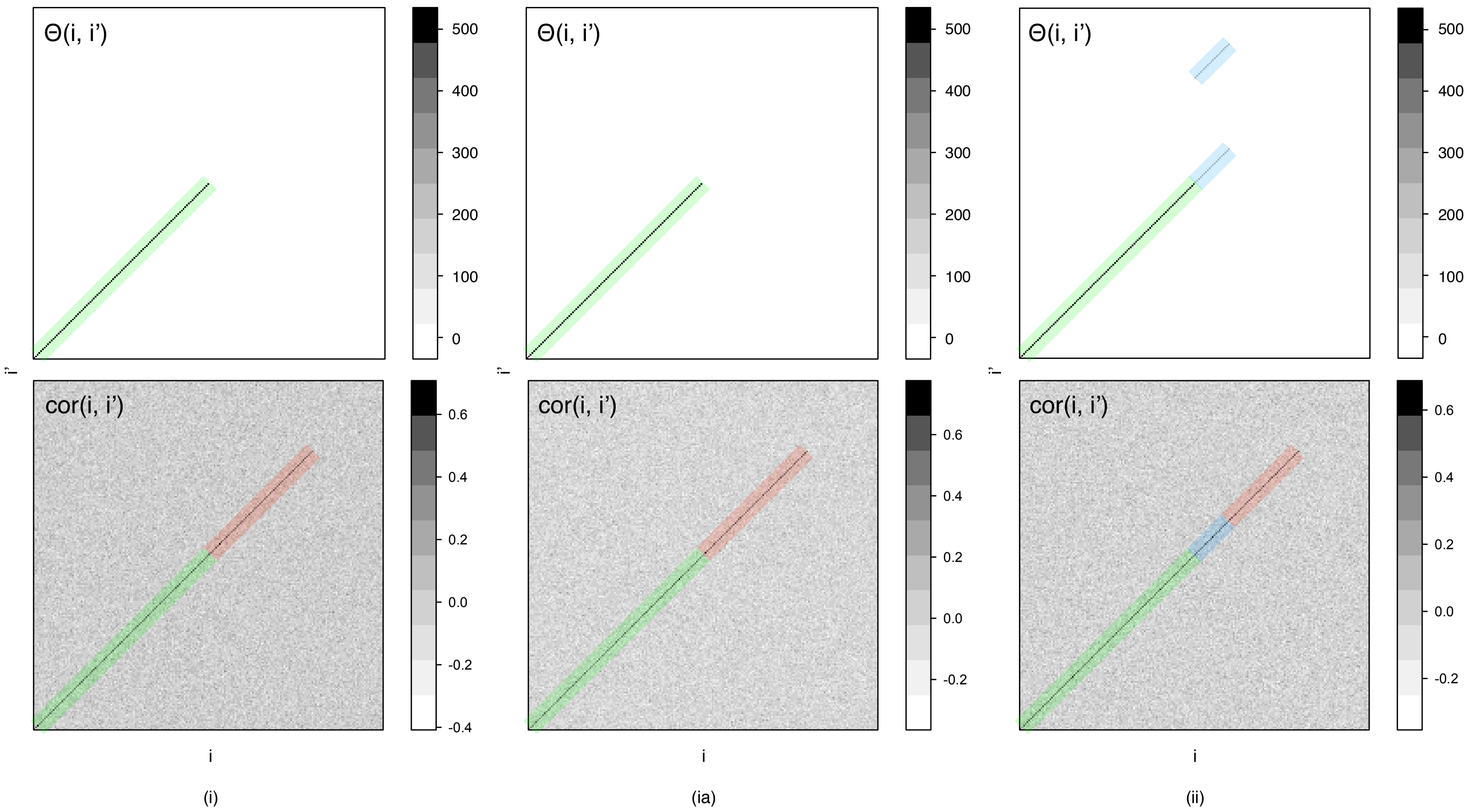

Figure S3: Matrix of vertex similarities $\Theta(i, i')$ (top) and matrix of correlations between the edge weights of vertices i in G and i' in G' (correlation of i'th column of A and i''th column of A', $cor(i, i')$, bottom) for the scenarios (i) and (ii) and network size $N = 200$. The optimal alignment of the two networks aligns the n-th vertex of G to the n-th vertex of G'. Half of the diagonal terms represents orthologous vertices with both vertex and topological similarity (highlighted in green). In scenario (i), the other 30% of vertices i in G have no vertex similarity but strong edge similarity (analogs, highlighted in red). In scenario (ii), 10% of the vertices in the network G (highlighted in blue) have two homologous vertices in the other network G', one of them with a strong topological match (the true ortholog) and the other with no match (the spurious ortholog). Scenario (ia) differs from scenario (i) in the underlying distribution of edge weights: in (i) the uniform distribution is used, while in (ia) the values are drawn from the normal distribution.

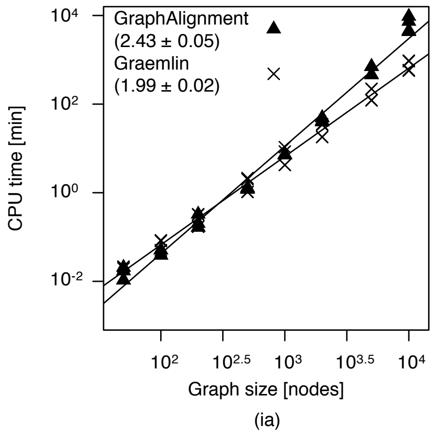

Figure S4: Computational complexity of the *GraphAlignment* and *Græmlin* algorithms in scenario (ia) with the edge weights drawn from the normal distribution.

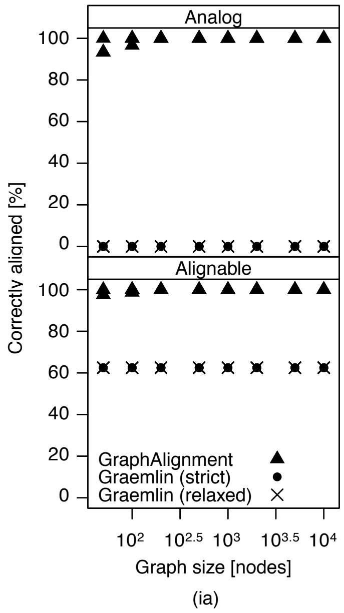

Figure S5: Accuracy of *GraphAlignment* and *Græmlin* in scenario (ia). While *GraphAlignment* aligns a large proportion or all analogous vertices, *Græmlin* aligns only the orthologous vertices with both vertex and topological similarity and no other vertices. The proportion of 62.5% corresponds to the fraction of those orthologs (50% of all vertices) among all orthologs (80% of all vertices).

Table S1: *GraphAlignment* and *Græmlin* performance on empirical bio-molecular networks. Gene co-expression networks (*continued*).

**Comparison**	***Escherichia coli vs. Shewanella oneidensis***		
**Algorithm**	*Graph-Alignment*	*Græmlin*	*Blast BBH*
**NA**	946	851	792
**NC**	537	505 (611)	604
**NO**	627	627	627
**NC / NA [%]**	56.8	59.3 (71.8)	76.3
**NC / NO [%]**	85.7	80.5 (97.5)	96.3
**Edge / vertex score**	4533 / 5082	-	-

See Table 2 of the main text for details.
